# Assessment of Intra-Abdominal Pressure with a Novel Continuous Bladder Pressure Monitor—A Clinical Validation Study

**DOI:** 10.3390/life13020384

**Published:** 2023-01-30

**Authors:** Liat Iacubovici, Dana Karol, Yuval Baar, Avi Beri, Haim Herzberg, Shiri Zarour, Or Goren, Barak Cohen

**Affiliations:** 1 Division of Anesthesia, Intensive Care, and Pain, Tel-Aviv Medical Center, Tel-Aviv University, Tel-Aviv 6423906, Israel; 2 Urology Department, Tel-Aviv Medical Center, Tel-Aviv University, Tel-Aviv 6423906, Israel; 3 Outcomes Research Consortium, Cleveland Clinic, Cleveland, OH 44195, USA

**Keywords:** intra-abdominal hypertension, abdominal compartment syndrome, intra-abdominal pressure monitoring, foley manometer

## Abstract

Introduction: Intra-abdominal hypertension and the resulting abdominal compartment syndrome are serious complications of severely ill patients. Diagnosis requires an intra-abdominal pressure (IAP) measurement, which is currently cumbersome and underused. We aimed to test the accuracy of a novel continuous IAP monitor. Methods: Adults having laparoscopic surgery and requiring urinary catheter intra-operatively were recruited to this single-arm validation study. IAP measurements using the novel monitor and a gold-standard foley manometer were compared. After anesthesia induction, a pneumoperitoneum was induced through a laparoscopic insufflator, and five randomly pre-defined pressures (between 5 and 25 mmHg) were achieved and simultaneously measured via both methods in each participant. Measurements were compared using Bland–Altman analysis. Results: In total, 29 participants completed the study and provided 144 distinct pairs of pressure measurements that were analyzed. A positive correlation between the two methods was found (R^2^ = 0.93). There was good agreement between the methods, with a mean bias (95% CI) of −0.4 (−0.6, −0.1) mmHg and a standard deviation of 1.3 mmHg, which was statistically significant but of no clinical importance. The limits of agreement (where 95% of the differences are expected to fall) were −2.9 and 2.2 mmHg. The proportional error was statistically insignificant (*p* = 0.85), suggesting a constant agreement between the methods across the range of values tested. The percentage error was 10.7%. Conclusions: Continuous IAP measurements using the novel monitor performed well in the clinical setup of controlled intra-abdominal hypertension across the evaluated range of pressures. Further studies should expand the range to more pathological values.

## 1. Introduction

Intra-abdominal hypertension (IAH) and abdominal compartment syndrome (ACS) are serious, life-threatening conditions [1,2,3]. The incidence of IAH and ACS in patients admitted to the intensive care unit (ICU) during the first week of stay is reported to be as high as 59% and 8%, respectively [2,4]. Associated mortality in ICU patients ranges from 25% to over 75%, depending on the underlying pathology and the corrective measures taken [5,6]. An accurate measurement of intra-abdominal pressure (IAP) is especially important in patients at risk since the early detection and management of IAH can potentially allow intervention and the prevention of ACS.

IAP values up to 5–7 mmHg are considered normal, but higher values are observed in ICU patients [2,7]. The diagnosis of IAH requires repeated measurements above 12 mmHg, while ACS is defined by repeated measurements above 20 mmHg accompanied by new organ failure [7,8].

The clinical assessment of IAP is unreliable with sensitivity of 40–60% [2,4,7]. IAP is therefore commonly estimated based on urinary bladder pressure [9]. This is measured using a manometer connected end-to-side to a urinary catheter, allowing the intermittent measurement of bladder pressure while temporarily impeding urinary flow. Some systems allow the use of a pressure sensor by introducing a needle into the system to allow pressure measurement, while others use a fluid column to measure pressure mechanically. Measuring the IAP via intra-bladder pressure is simple, reliable, reproducible, and minimally invasive since most relevant patients have a urinary catheter for other reasons [2,8]. Nonetheless, results are highly dependent on the patient position, zero-point definition, and user expertise. Other concerns include the need to repeatedly obstruct urine flow and the risk of iatrogenic urinary tract infections.

A novel urinary flow-meter (Serenno Medical Ltd., Yokne’am Illit, Israel) is able to continuously estimate urinary bladder pressure without the interruption of urinary flow. The technology is based on a sensor serially connected between the urinary catheter and the collection bag. Repeated aspirations of small, constant volumes of fluid allow the measurement of flow and an estimation of pressure while urine flow remains uninterrupted. The system can therefore potentially detect IAH earlier, while avoiding disconnections and reducing infection risk. However, the system’s accuracy, although tested in laboratory settings, has not been properly validated in clinical settings to date [10].

We therefore aimed to evaluate the accuracy of the novel flow-meter in the assessment of bladder pressure and compare it to the gold-standard fluid-column foley manometer. We hypothesized that the bias, calculated as the mean difference between the study device and the foley manometer, would not exceed 1 mmHg as recommended by the Abdominal Compartment Society (formerly known as the world society of abdominal compartment syndrome, WSACS) guidelines [11].

## 2. Materials and Methods

This single-center validation observational study was approved by the Tel-Aviv Medical Center institutional review board (IRB TLV-21-0406, chairperson prof. Berliner, date of approval 27 October 2021) and was electronically registered in the American National Institution of Health registry prior to patient enrollment (clinicaltrials.gov NCT05121454). Eligible patients were approached for potential participation and were enrolled after providing written informed consent.

We approached adults undergoing laparoscopic surgery that required the intra-operative use of urinary catheters between November 2021 and March 2022 in the Tel-Aviv Medical Center (Tel-Aviv, Israel). Patients with structural urologic or renal disturbances, pregnant women, and patients unable to provide informed consent were excluded.

**Figure 1:**
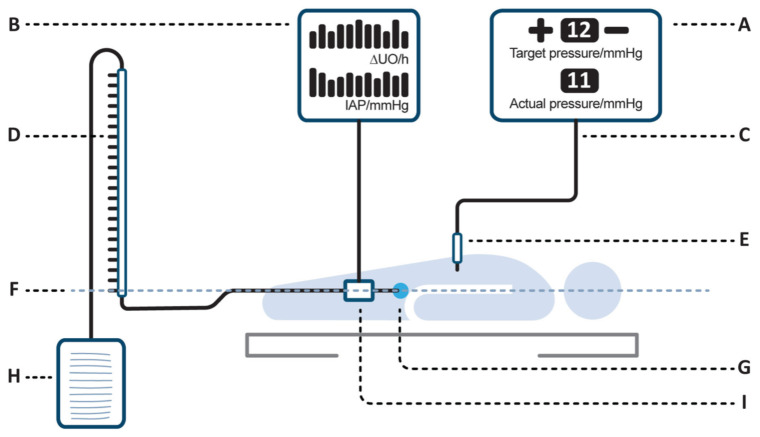
Study system design (not to scale). A—Insufflator—allows setting a pre-defined abdominal pressure during the study. B—Study device control unit—mounted on a pole or bed-rail. Can be placed anywhere near the bed or move with the patient. C—Tube from CO_2_ insufflator to the surgical port. D—Classic fluid column manometer measures intra-vesical pressure via a Foley catheter. E—Surgical port into the peritoneal cavity. F—Mid-axillary line (reference level). G—Urinary bladder. H—Urine collection bag. I—Study device disposable measurement unit, located between the Foley catheter and urine bag, and transmitting pressures to the controller.

A urinary catheter was inserted after the induction of general anesthesia. The catheter was then serially connected to the study device, followed by a classic fluid–column manometer and a urine collection bag. If the initial urine volume was not sufficient to fill the fluid manometer, up to 25 mL of saline was added to the system in a sterile fashion. Once the peritoneal cavity was insufflated with carbon dioxide, the study device started to continuously record bladder pressures and a concealed envelope containing 5 random target pressures between 5 and 25 mmHg was opened. The laparoscopic insufflator was set to reach and hold each pre-defined pressure, and bladder pressures were recorded simultaneously from the two systems (namely the study device and the fluid manometer). Each target pressure was kept for 30 s prior to recording to allow pressure stabilization. Once all 5 measurements were completed, the study device and fluid manometer were disconnected, the bladder catheter was directly connected to the urine collection bag, and the surgical procedure was started. The study setup is illustrated in Figure 1. Each study measurement required about 1 min, and all measurements were completed in approximately 5 min. To reduce potential measurement bias, the study device recorded pressures based on an internal memory, so researchers were kept blinded to the device measurements. To assure paired recordings, the device was equipped with a button that was pressed when values were simultaneously recorded from the fluid manometer. Each press of the button added a mark to the continuous pressure recordings, and only these corresponding values were considered for the analysis.

## 3. Statistical Analysis

Baseline demographic, anthropomorphic, and medical variables, as well as details of the surgical and anesthetic procedures were summarized with descriptive statistics.

Each pair of measurements (study device and fluid column manometer) was first plotted on a scatter-plot and analyzed using the Pearson correlation. We then used the Bland–Altman analysis to test the level of agreement between the bladder pressures recorded by the study device and those recorded by the fluid manometer. We pre-defined the maximal allowed difference (delta) to 3 mmHg, which is in line with the standard values limits of 4 mmHg, as recommended by the Abdominal compartment society (formerly known as the WSACS) guidelines [11]. In order to conclude that the two measurement methods are in agreement, the 95% confidence interval around the mean difference (the limits of agreement around the bias) would have to fall within the maximal allowed difference. SPSS Statistics for Windows, Version 27 (IBM, Armonk, NY, USA: IBM Corp.), was used for all analyses.

## 4. Results

**Figure 2:**
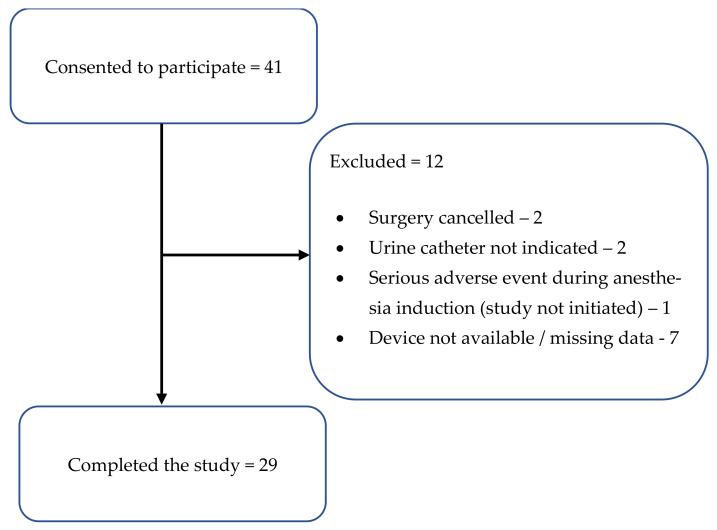
Study flow-chart.

In total, 41 patients consented to participate. After excluding cases that were canceled or did not require the insertion of a urinary catheter and removing cases with fundamental data missing due to technical problems (e.g., no pressure recording on the study device internal memory), 29 patients remained in the analysis, providing 144 distinct pairs of pressure measurements (Figure 2). Most participants were men (66% vs. 34% women), the median [interquartile range (IQR)] age was 66 [53, 77] years, and the median [IQR] American Society of Anesthesiologists’ physical status (ASA-PS) score was 2 [2, 2]. Most surgeries were kidney or prostate resections, and some were gynecologic or gallbladder surgeries. However, all study measurements were performed in the supine position, before the surgical procedure began. Other baseline characteristics are detailed in Table 1. The mean (SD) opening pressure was 2.6 (4.1) mmHg. Five participants required an initial volume bolus into the urine catheter in order to sufficiently fill the fluid manometer.

**Figure 3:**
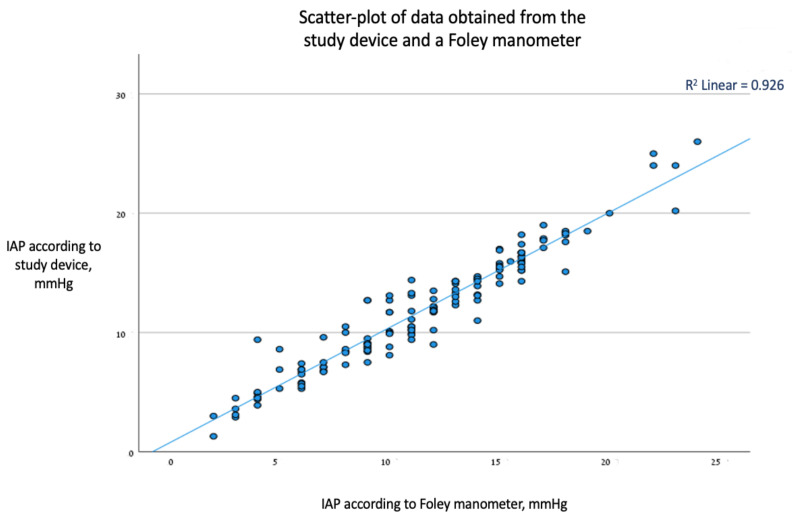
Scatter-plot of acceptance between the two measurement methods.

We performed Pearson’s correlation analysis and found an overall positive correlation between the pressure readings from the novel continuous bladder pressure monitor and those of the gold-standard fluid manometer, with a statistically significant positive R of 0.962 (*p* < 0.01). R^2^ was 0.93. The correlation formula is IBP_Foley_ = 0.14 + 0.96 × IBP_study device_ (Figure 3).

**Figure 4:**
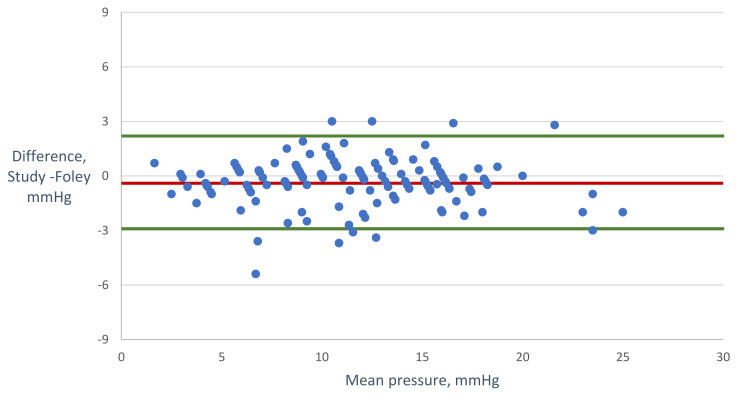
Bland–Altman analysis. The Y-axis shows the difference between measurements made by the study device minus those of the foley manometer, and the X-axis presents the means of measurements via both methods. The red line represents the mean bias (-0.4 mmHg), and green lines represent the upper and lower limits of agreement (2.2 and -2.9 mmHg, respectively).

Bland–Altman analysis revealed good agreement between the readings from the two measurement devices, with a bias (95% CI) of −0.4 (−0.6, −0.1) mmHg (*p* = 0.01) and 95% limits of agreement (2 standard deviations around the mean difference) of −2.9 and 2.2 mmHg (Figure 4). The fixed bias of −0.4 mmHg with a standard deviation of 1.3 mmHg was statistically significant, although probably of no clinical importance. Additionally, we did not detect proportional bias (a correlation coefficient of 0.04, *p* = 0.85). The percentage error was 10.7%.

## 5. Discussion

In this validation study, we aimed to test the accuracy of a novel urinary flowmeter in measuring bladder pressure, compared to a gold-standard fluid-column Foley manometer. We found good agreement between the study device and the gold-standard method in the range of 5 and 25 mmHg. The absolute difference (system bias) between the methods was 0.4 mmHg, which probably carries no clinical importance. Importantly, both the bias and the confidence interval around it fall well within the Abdominal Compartment Society recommendations for validation of new IAP monitoring devices [11].

High IAP may go unrecognized as it primarily affects patients who are already severely ill and whose organ dysfunction may be incorrectly attributed to the progression of their primary condition [4,12,13]. Since early recognition and treatment may improve organ dysfunction, a high index of suspicion and frequent monitoring are of utmost importance in the appropriate clinical setup [12,14]. An international survey among physicians demonstrated high awareness of IAH and ACS but found considerable inconsistency in IAP monitoring and treatment [15].

The Abdominal Compartment Society recommends the use of Foley manometers as the gold-standard for IAP measurements. Nonetheless, available methods are cumbersome and are therefore of limited use. The main disadvantages of the common commercial solutions are the need to perform the measurements manually, the repeated interruptions to urine flow, the repeated introduction of saline or urine back to the bladder, and sometimes the need to repeatedly disconnect the urine collection system, potentially disrupting system sterility.

The novel system we tested (Serenno Medical Ltd., Yokne’am Illit, Israel) aimed at accurately and continuously measuring urine output. Yet another capability of the system is to continuously measure bladder pressure without interrupting urine flow. The system therefore introduces several potential advantages over existing IAP monitoring systems, including less user-dependence, a decrease in the nursing workload, potentially improved accuracy, continuous (rather than intermittent) monitoring with no interruption to urine flow, and the lack of sterility disruption.

To the best of our knowledge, this is the first study that reports on continuous intra-abdominal pressure monitoring in a clinical setting. As discussed previously, the implementation of continuous IAP devices may lead to the earlier detection of high IAP and ACS and timely intervention in high-risk ICU patients, thus improving medical care. Early detection of high IAP and ACS may also lead to improved economical outcome by reducing medical costs of a delayed diagnosis and its complications [16].

We chose to test the accuracy of the system in a controlled clinical setup of increased intra-abdominal pressure—adults undergoing laparoscopic surgery. We were therefore able to control and modify intra-abdominal pressures produced by the laparoscopic CO_2_ insufflator and to compare the pressures recorded by the study device to those captured by a commercial Foley manometer. Since all measurements were performed shortly after the induction of general anesthesia but before the actual surgery began, patients were well-paralyzed and ventilation was mechanically-controlled; thus, diaphragmatic activity and abdominal muscle tension did not interfere with measurements. Another strength of our study is that researchers were blinded to the device recordings, as it was set not to display any values in real time. Researchers only recorded values from the control measurements, and all comparisons were performed during the statistical analysis. We believe that this study design reduced potential measurement bias.

In seven cases, no interpretable data were available due to technical issues (mostly battery life or malfunction of disposables). These issues need to be addressed by the manufacturer before the system becomes completely commercial. However, since these events occurred at random, we believe that they did not present any bias to the study results.

The main limitation of our study is that the range of pressures used to test the system corresponds with normal IAP values, as well as pressures found in patients with IAH, but below the threshold of severe IAH—presumably the most important clinical application. We were limited both by the ethical concerns of exposing surgical patients to high pressures, even if for a short period, and by the technical limitations of the commercial insufflator. Our conclusions are therefore primarily valid for the pressure range tested in the study, and one could argue that at higher pressures, the system might perform differently. Nonetheless, we did not find any signal of proportional bias, meaning that within the range tested, the system performed equally well across the various pressures. It is therefore reasonable to assume that the correlation extends to somewhat higher pressures.

Other than sex, weight, height, and BMI, no other anthropomorphic data were collected in this study. In addition, we are unable to calculate the CO_2_ volume of insufflation. Such data may allow an exploration of compliance in the abdominal compartment and may be interesting to explore in future studies of IAH and ACS.

In conclusion, in the IAP range of 5 to 25 mmHg, we found that the Serenno Medical continuous bladder pressure monitor correlates well with the gold-standard method for IAP measurements. This might allow its use as a continuous intra-bladder pressure monitor in patients at risk of intra-abdominal hypertension. Furthermore, we believe that this study may pave the way for future research and an understanding of the implications of continuous intra-abdominal pressure measurements. Future studies should expand the range to more pathological values.

## Figures and Tables

**Table 1:** Main patient characteristics.

Parameter	
Sex, female	10 (34%)
Age, years	66 [53, 73]
Weight, Kg	79 (14)
BMI	27.4 (4.7)
ASA-PS	2 [2, 2]
Current smokers	6 (21%)

Values are presented as the N (%), mean (standard deviation), or median [interquartile range], as appropriate. ASA-PS, American Society of Anesthesiologists physical status score; BMI, body mass index.

## Data Availability

The data presented in this study are available on request from the corresponding author. The data are not publicly available due to restriction of the IRB.

## References

[1] Al-Abassi A.A., al Saadi A.S., Ahmed F. (2018). Is Intra-Bladder Pressure Measurement a Reliable Indicator for Raised Intra-Abdominal Pressure? A Prospective Comparative Study. BMC Anesthesiol..

[2] Milanesi R., Caregnato R.C.A. (2016). Intra-Abdominal Pressure: An Integrative Review. Einstein.

[3] Parsak C.K., Seydaoglu G., Sakman G., Acarturk T.O., Karakoc E., Hanta I., Alparslan A.H., Satar S. (2008). Abdominal Compartment Syndrome: Current Problems and New Strategies. World J. Surg..

[4] De Laet I.E., Ravyts M., Vidts W., Valk J., de Waele J.J., Malbrain M.L.N.G. (2008). Current Insights in Intra-Abdominal Hypertension and Abdominal Compartment Syndrome: Open the Abdomen and Keep It Open!. Langenbecks Arch. Surg..

[5] Bodnar Z. (2019). Polycompartment Syndrome—Intra-Abdominal Pressure Measurement. Anaesthesiol. Intensive Ther..

[6] Popescu G.A., Bara T., Rad P. (2018). Abdominal Compartment Syndrome as a Multidisciplinary Challenge. A Literature Review. J. Crit. Care Med..

[7] Kirkpatrick A.W., Roberts D.J., de Waele J., Jaeschke R., Malbrain M.L.N.G., de Keulenaer B., Duchesne J., Bjorck M., Leppaniemi A., Ejike J.C. (2013). Intra-Abdominal Hypertension and the Abdominal Compartment Syndrome: Updated Consensus Definitions and Clinical Practice Guidelines from the World Society of the Abdominal Compartment Syndrome. Intensive Care Med..

[8] Van der Steeg H., Van Akkeren J.P., Houterman S., Roumen R.M.H. (2009). Validation of the Urine Column Measurement as an Estimation of the Intra-Abdominal Pressure. Intensive Care Med..

[9] Malbrain M.L.N.G. (2004). Different Techniques to Measure Intra-Abdominal Pressura (IAP): Time for a Critical Re-Appraisal. Intensive Care Med..

[10] Tayebi S., Wise R., Pourkazemi A., Stiens J., Malbrain M.L.N.G. (2022). Pre-Clinical Validation of A Novel Continuous Intra-Abdominal Pressure Measurement Equipment (SERENNO). Life.

[11] De Waele J., Cheatham M., Malbrain M., Kirkpatrick A., Sugrue M., Balogh Z., Ivatury R., De Keulenaer B. (2009). Recommendations for research from the international conference of experts on intra-abdominal hypertension and abdominal compartment syndrome. Acta Clin. Belg..

[12] Tiwari A.R., Pandya J.S. (2016). Study of the Occurrence of Intra-Abdominal Hypertension and Abdominal Compartment Syndrome in Patients of Blunt Abdominal Trauma and Its Correlation with the Clinical Outcome in the above Patients. World J. Emerg. Surg..

[13] Khanna A.K., Minear S., Kurz A., Moll V., Stanton K., Essakalli L., Prabhakar A., Harris L.C., Sweatt N., Flores K. (2023). Intra-Abdominal Hypertension in Cardiac Surgery Patients: A Multicenter Observational Sub-Study of the Accuryn Registry. J. Clin. Monit. Comput..

[14] Malbrain M.L.N.G., Cheatham M.L., Kirkpatrick A., Sugrue M., Parr M., de Waele J., Balogh Z., Leppäniemi A., Olvera C., Ivatury R. (2006). Results from the International Conference of Experts on Intra-Abdominal Hypertension and Abdominal Compartment Syndrome. I. Definitions. Intensive Care Med..

[15] Wise R., Rodseth R., Blaser A.R., Roberts D.J., de Waele J.J., Kirkpatrick A.W., de Keulenaer B.L., Malbrain M.L.N.G. (2019). Awareness and Knowledge of Intra-Abdominal Hypertension and Abdominal Compartment Syndrome: Results of a Repeat, International, Cross-Sectional Survey. Anaesthesiol. Intensive Ther..

[16] Ney J.P., Moll V., Kimball E.J. (2022). Urinary Catheter Monitoring of Intra-Abdominal Pressure after Major Abdominal Surgery, a Cost-Benefit Analysis. J. Med. Econ..

